# Complex Dielectric Permittivity Spectra of Rapeseed in the 20 MHz–3 GHz Frequency Range

**DOI:** 10.3390/ma15144844

**Published:** 2022-07-12

**Authors:** Marcin Kafarski, Agnieszka Szypłowska, Jacek Majcher, Andrzej Wilczek, Arkadiusz Lewandowski, Zuzana Hlaváčová, Wojciech Skierucha

**Affiliations:** 1 Institute of Agrophysics, Polish Academy of Sciences, 20-290 Lublin, Poland; m.kafarski@ipan.lublin.pl (M.K.); a.wilczek@ipan.lublin.pl (A.W.); w.skierucha@ipan.lublin.pl (W.S.); 2 Department of Electrical Engineering and Electrotechnologies, Lublin University of Technology, 20-618 Lublin, Poland; j.majcher@pollub.pl; 3 Institute of Electronic Systems, Warsaw University of Technology, 00-665 Warsaw, Poland; a.lewandowski@elka.pw.edu.pl; 4 Department of Physics, Faculty of Engineering, Slovak University of Agriculture in Nitra, SK-949 76 Nitra, Slovakia; zuzana.hlavacova@uniag.sk

**Keywords:** granular materials, dielectric spectroscopy, radio and microwave measurements, moisture content, rapeseed

## Abstract

Rapeseed is one of the most important sources of vegetable oil worldwide. Knowledge of the dielectric properties of rapeseed may be beneficial for moisture content determination and the optimization of microwave treatment processes. The aim of this research was to examine the complex dielectric permittivity spectra of rapeseed of moisture content from 8.3% to 16.1%. The measurements were performed in the 20 MHz–3 GHz frequency range with the use of a vector network analyzer and a coaxial transmission-line cell. The real part of dielectric permittivity significantly depended on the water content of the seeds. The obtained spectra were modeled with the use of a three-pole Debye model with bulk electrical conductivity. Because the highest-frequency pole was found near the high-frequency measurement band limit, the spectra were additionally modeled with the use of an approximate ABC model with two in-band Debye poles. The determined model parameters were found to be highly dependent on the water content of the seeds. The relations between these parameters and water content were analyzed.

## 1. Introduction

Relative dielectric permittivity $\varepsilon^{*}$ is generally a complex physical quantity:

(1) $$\varepsilon^{*}=\varepsilon^{\prime }-j\varepsilon^{\prime \prime },$$

where $\varepsilon^{\prime }$ is the real part of the dielectric permittivity, describing the polarization of the given medium under the influence of an external electric field, $\varepsilon^{\prime \prime }$ is the imaginary part of dielectric permittivity, describing the energy losses in the medium, and *j* is the imaginary unit. In complex materials of biological origin, $\varepsilon^{*}$ depends on frequency *f* of an external electric field and may exhibit significant dielectric dispersion in the MHz–GHz frequency range due to multiple dielectric relaxation mechanisms, such as free and bound water relaxation, various interphase phenomena such as the Maxwell–Wagner effect, as well as the rotations and hydration of large organic molecules [1]. The investigation of dielectric properties of crops and various food products has been conducted for many decades. Knowledge of complex relative dielectric permittivity of these products may be advantageous for developing and improving moisture content determination methods, optimizing dielectric processes such as heating and drying, as well as in the determination of important quality factors of given foods [2,3,4,5].

Rapeseed (*Brassica napus* L.) is one of the most important oil-producing crops in the world [6]. Its high nutritional value stems from the presence of fatty acids, which include a relatively high amount of omega-3 acids, as well as proteins and other chemical compounds [7]. After oil extraction, rapeseed meal is commonly utilized as animal feed and can be used as a source of antioxidants [8]. The dielectric pretreatment of rapeseed was found to increase the oil extraction yield [9] and decrease the amount of toxic substances in the meal [10]. Knowledge of the dielectric properties of rapeseed is important for the development of microwave treatments and processing [11].

The determination and control of the moisture content of rapeseed is also important—it is recommended that the seeds are stored at a moisture content no higher than 10%, as excessive moisture may cause undesirable biochemical activity, self-heating and degradation of important nutritional compounds [12]. The moisture content of rapeseed can be assessed with moisture analyzers based on weighing and drying the seeds at an elevated temperature. There are also devices that utilize various electrical techniques involving, e.g., the measurement of resistance or capacitance, which enable rapid testing [13]. Research on the measurement methods of the moisture of individual seeds, which could be especially useful before harvest, is also conducted [14]. Recent advancements in dielectric measurement methods are promising in nondestructive moisture monitoring [15]. Therefore, knowledge of the dielectric properties of rapeseed may be useful for the development and improvement of the moisture content monitoring of seeds.

Macroscopically, a sample of rapeseeds subjected to dielectric measurements consists of approximately spherical seeds and air present in pores and spaces between the seeds. Therefore, it can be regarded as a two-phase porous material. However, each seed is comprised of the hull and germ. On a molecular level, it has a rich chemical composition and can possess a variable amount of water inside the seed, including hydration water bound to large organic molecules. The dielectric permittivity measurements of such complex inhomogeneous materials is difficult due to the necessity of ensuring the large measurement volume of the material under test, which poses technical difficulties connected to the propagation of electromagnetic waves, especially at microwave frequencies. In order to circumvent this problem, in [16] rapeseeds were ground and then measured in a fixture equipped with an open-ended probe in the frequency range from 10 MHz to 3 GHz at temperatures from 20 to 60 °C. Paper [11] presents the dielectric characterization of rapeseeds also in powder form at 8.93 GHz at various packing fractions at room temperature. The dielectric properties of rapeseed oil were also measured at various radio and microwave frequencies [17,18]. In [19], $\varepsilon^{*}$ of whole seeds was measured at 5–30 MHz in 30–80 °C temperature range with the use of an LCR meter and an Agilent dielectric test fixture which was a parallel-plate capacitor configured for 16.4 mL of measured material volume. The present study aimed to perform the dielectric characterization of whole rapeseeds in the frequency range from 20 MHz to 3 GHz.

The goal of this research was to investigate the complex permittivity spectra of rapeseed samples consisting of whole seeds of moisture content ranging from 8.3% to 16.1% (from 0.083 to 0.161 g $g^{-1}$ calculated on a wet mass basis). The selected moisture range is typical for seeds during harvest. The measurements were performed in coaxial transmission-line cells of large diameter [20], which enabled the measurement of whole seeds without any special preparation such as grinding. The relations between moisture content and dielectric permittivity were established. Furthermore, two dielectric models were fitted to the spectra. The obtained parameters were correlated with the moisture content of the seeds and the appropriate relations were determined.

## 2. Materials and Methods

### 2.1. Preparation of the Material under Test

For the measurements, rapeseed of the Bellevue cultivar was used. The seeds were harvested in 2021 on a local farm near Lublin, Poland. At first, the seeds were sieved in order to ensure that the diameter of the seeds prepared for the experiment were relatively uniform in the range from 1.7 to 2 mm. Ten samples of moisture content θ in the specified range were prepared by mixing the seeds with predefined amounts of water and placing them in air-tight containers. Then, the samples were conditioned for 24 h in a refrigerator at the temperature of 5–8 °C. During this time, the containers were opened and the material was stirred every three hours. After the conditioning process, the resultant moisture content of the prepared samples was determined by taking the smaller samples from the containers and drying them in a RADWAG WPS 30S moisture analyzer (Radom, Poland). The values of θ were calculated from the ratio of the mass of the evaporated water and the initial mass of the sample and expressed in g $g^{-1}$. Alternatively, θ may be expressed in % by multiplying the values by 100%. The prepared samples were stored in closed containers in a refrigerator, in order to keep the moisture content stable and prevent germination.

### 2.2. Dielectric Spectra Measurement

Complex dielectric permittivity spectra of the samples were measured by a six-cell coaxial transmission-line system connected to a Copper Mountain R60 single-channel vector network analyzer (VNA) (Indianapolis, IN, USA). The experimental setup was designed and previously used for dielectric measurements of soil samples. The system consisted of six acid-resistant stainless steel sample cells, each of which was connected at one port to an electronic calibration unit (ECU) with four states and at the other port connected to an RF switch, which was then connected to the VNA. In each cell, the material under test was placed in a chamber formed between the outer conductor of 38.8 mm in diameter and the inner conductor of 16.9 mm in diameter. The samples were kept in place by plastic supports equipped with o-rings to prevent evaporation. The position of the supports was adjustable and enabled the measurement of samples of volumes between 34 and 41 ${\mathrm{cm}}^{3}$. Each channel of the system was calibrated with the use of the ECU and an empty cell. The complex dielectric permittivity spectra of the samples under test were calculated from the measured complex reflection coefficients with the use of a nonlinear least squares algorithm. The details of the measurement system in a single-channel version, the calibration procedures and measurement accuracy verification were presented in [20].

Containers with samples destined for testing on a given day were removed from the refrigerator at least two hours before the measurement and kept at room temperature. This ensured that the temperature of the seeds under test was 24.7 ± 1.7 °C. For each moisture content, three seed portions were taken out of the sample container and placed in their respective measurement cells. The average density of the tested samples was 0.74 g ${\mathrm{cm}}^{-3}$ with a standard deviation of 0.02 g ${\mathrm{cm}}^{-3}$. All measurements were performed in an air-conditioned laboratory at 25 °C. In total, 30 samples were tested. However, due to an experimental protocol error discovered after the measurements, one sample of θ=0.142 g $g^{-1}$ was rejected, which resulted in 29 dielectric spectra taken for further analysis.

### 2.3. Dielectric Spectra Modeling and Analysis

After the dielectric spectra were obtained, the relations between the real part of dielectric permittivity at given frequencies and moisture content were modeled by an equation linear with respect to the square root of $\varepsilon^{\prime }$:

(2) $$\sqrt{\varepsilon^{\prime }}=a\theta +b,$$

where θ was the moisture content expressed in g $g^{-1}$. An equation of this form, reformulated as a $\theta (\sqrt{\varepsilon^{\prime }})$ relation, is a popular formula used for soil moisture determination by dielectric methods [21,22]. The values of the parameters *a* and *b* of Equation (2) were established after fitting the equation to the experimental data at each frequency independently with the use of the *fitlm* function in the Matlab (2019a) [23] environment. The frequency dependence of the *a* and *b* parameters was modeled by a fourth-order polynomial calculated with respect to the natural logarithm of the frequency *f*, according to the following equation:

(3) $$y=c_{0}+c_{1}\ln f+c_{2}\ln^{2}f+c_{3}\ln^{3}f+c_{4}\ln^{4}f,$$

where *y* stands for either *a* or *b*. The fitting was also performed with the Matlab *fitlm* function.

In order to investigate the dielectric relaxation of the measured samples, the obtained spectra were modeled with the use of a three-pole Debye model (labeled ‘3D’ in the present paper) with the added bulk electrical conductivity term:

(4) $$\varepsilon^{*}\left(f\right)=\varepsilon_{\infty }+\sum_{n=1}^{3}\frac{\Delta \varepsilon_{n}}{1+j2\pi f\tau_{n}}+\frac{\sigma_{b}}{j2\pi f\varepsilon_{0}},$$

where $\varepsilon_{\infty }$ is the high-frequency-limit permittivity, $\Delta \varepsilon_{n}$ and $\tau_{n}$ are relaxation amplitude and time of an *n*-th pole, respectively, $\sigma_{b}$ is bulk electrical conductivity and $\varepsilon_{0}$ is the absolute dielectric permittivity of vacuum. The relaxation frequency $f_{n}$ of the *n*-th pole can be calculated from the relaxation time with the use of the following formula:

(5) $$f_{n}=\frac{1}{2\pi \tau_{n}}.$$

Because the upper frequency limit of the measurement setup was 3 GHz, which may not be enough to accurately extract the high-frequency free-water-dipole dielectric relaxation pole, the ABC model introduced in [24], but with two Debye-type poles, was also fitted to the spectra. In the present paper, this model was labeled ‘2D-ABC’. The form of the model is as follows:

(6) $$\varepsilon^{*}\left(f\right)=A-j2\pi fB-{\left(2\pi f\right)}^{2}C+\sum_{n=1}^{2}\frac{\Delta \varepsilon_{n}}{1+j2\pi f\tau_{n}}+\frac{\sigma_{b}}{j2\pi f\varepsilon_{0}},$$

which was also fitted to the spectra. *A*, *B* and *C* parameters were used to approximate the high-frequency pole. After fitting, the high-frequency pole relaxation frequency $f_{3}$, amplitude $\Delta \varepsilon_{3}$ and high-frequency limit $\varepsilon_{\infty }$ can be recovered with the use of the following formulas:

(7) $$A=\Delta \varepsilon_{3}+\varepsilon_{\infty },\;\;\;B=\frac{\Delta \varepsilon_{3}}{2\pi f_{3}},\;\;\;C=\frac{\Delta \varepsilon_{3}}{{\left(2\pi f_{3}\right)}^{2}}.$$

Both aforementioned models were fitted to the experimental data with the use of a Matlab procedure utilizing nonlinear-least-squares method.

Then, the dependence of the determined parameters of these models on the moisture contents of the seeds was examined. Three types of functions were used in order to model these relations:

1. Linear function:
  (8) y=ax+b,
2. Quadratic function:
  (9) $$y=ax^{2}+bx+c,$$
3. Segmented model consisting of two linear segments (introduced in [24]):
  (10) $$\left\{\begin{array}{cc} y=b_{s1}+a_{s1}x & x\leq x_{c}\\ y=b_{s1}+\left(a_{s1}-a_{s2}\right)x_{c}+a_{s2}x & x\geq x_{c} \end{array}\right.,$$
  where $x_{c}$ is the common point, in which both segments connect.

In the aforementioned equations of the segmented model, *x* was always the moisture content expressed in g $g^{-1}$. In each case, the quality of the fit for these relations was examined based on the calculated coefficient of determination $R^{2}$ and the root-mean-squared error RMSE. The fitting was executed with the use of a Matlab script.

## 3. Results and Discussion

### 3.1. Dielectric Spectra

**Figure 1:**
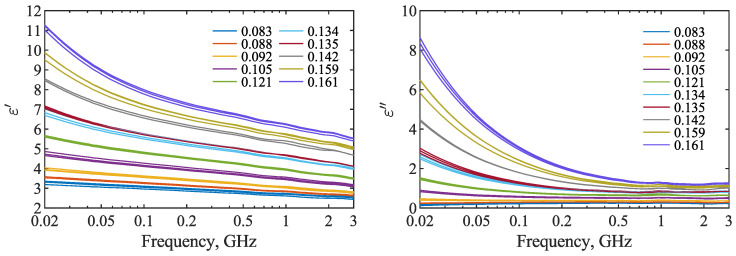
Spectra of the real (**left**) and imaginary (**right**) parts of the complex dielectric permittivity of all measured rapeseed samples of moisture content given in the legend (in g $g^{-1})$.

Dielectric spectra of all measured samples are presented in Figure 1. Samples of the same moisture content are represented by the same line color. In order to assess the spread of the spectra of samples of the same moisture content, the relative mean absolute deviation rMAD was calculated for each θ and at each frequency, separately for $\varepsilon^{\prime }$ and $\varepsilon^{\prime \prime }$. For $\varepsilon^{\prime }$, the values of rMAD did not exceed 2%, with the average value equal to only 0.95%. In the case of $\varepsilon^{\prime \prime }$, the highest value of rMAD=16.7% was observed at 20 MHz in the case of the lowest moisture content, for which the average value of $\varepsilon^{\prime \prime }$ was only 0.16. The average value of rMAD for $\varepsilon^{\prime \prime }$ was 2.2%.

**Figure 2:**
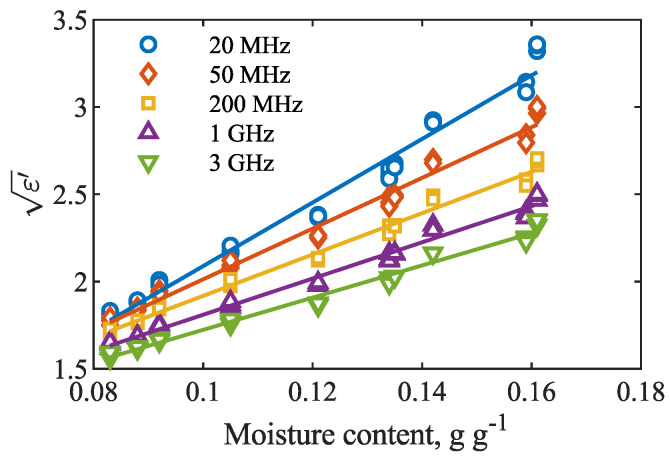
Relations between the square root of the real part of dielectric permittivity $\sqrt{\varepsilon^{\prime }}$ at selected frequencies and moisture content of rapeseed.

As expected, both real and imaginary parts of dielectric permittivity increased with the increase in moisture content of the seeds. The $\varepsilon^{\prime }\left(\theta \right)$ relation was modeled with the use of Equation (2). The results are presented in Figure 2. The fitting provided satisfactory results with $R^{2}\in \left(0.9788,0.9888\right)$ and RMSE∈(0.033,0.076), and the best fit was obtained at 660 MHz. The parameters of the fitted lines, root-mean-squared error RMSE and coefficient of determination $R^{2}$ of the fit at selected frequencies are given in Table 1.

**Figure 3:**
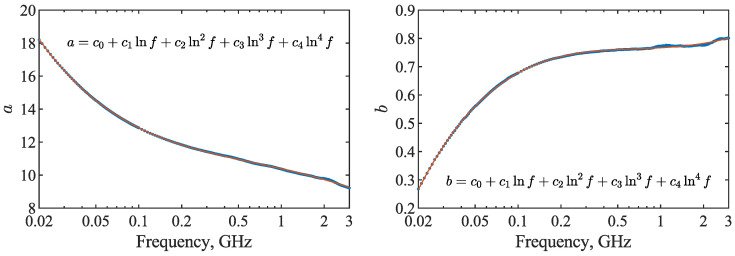
Slope *a* and intercept *b* (blue dots) of the $\sqrt{\varepsilon^{\prime }}\left(\theta \right)$ relation (Equation (2)) as functions of frequency. The frequency dependencies of the *a* and *b* parameters were modeled by a fourth-order polynomial (red lines) with respect to the natural logarithm of the frequency *f* expressed in GHz, according to Equation (3).

The frequency dependence of the *a* and *b* parameters of Equation (2) is depicted in Figure 3. The slope was found to be a descending function of the frequency, while the intercept was an ascending function of the frequency. It occurred that both relations were modeled by a fourth-order polynomial with respect to the natural logarithm of frequency (Equation (3)). The values of the coefficients obtained during fitting are presented in Table 2. The $R^{2}$ and RMSE of the fit are equal to 0.9998 and 0.028 for the *a* parameter, respectively, while in the case of the *b* parameter, the values of 0.9991 and 0.003 were obtained. Because of relatively high polynomial order, standard errors, *t*-statistics and *p*-values of the coefficients calculated by the *fitlm* function [25] are also provided in the table in order to show the significance of the parameters.

Direct comparison of the results obtained in the present study with existing literature is difficult due to the natural variability of seed properties depending on cultivar or field conditions such as sunlight and nutrient availability [26]. As a result, the physical and chemical properties of given seed samples may differ. In [19], the bulk density of rapeseed was lower than in the present study. Furthermore, measurements in [19] were conducted at higher temperatures, and temperature has a significant influence on the dielectric permittivity of rapeseed, as shown in [16,19]. Permittivity spectra in [16] were determined for compacted powder obtained from ground seeds. The density of the tested material was approximately between 1.04 and 1.08 g ${\mathrm{cm}}^{-3}$, which is significantly higher than the bulk density of seeds in the present study. As expected, because of a greater amount of air in samples consisting of whole seeds than in the compressed powder, both $\varepsilon^{\prime }$ and $\varepsilon^{\prime \prime }$ values are lower in the present study than in [16]. However, the general shapes of the permittivity spectra are quite similar, indicating the presence of a low-frequency relaxation mechanism below 50–100 MHz and a more-or-less steady decrease in $\varepsilon^{\prime }$ with frequency at higher frequencies. Visual assessment of the spectra from both works might hint at a possible presence of a middle-frequency relaxation at approximately or below 1 GHz. The dielectric relaxation in spectra obtained in the present study is assessed in the next section.

### 3.2. Dielectric Modeling and Analysis

**Figure 4:**
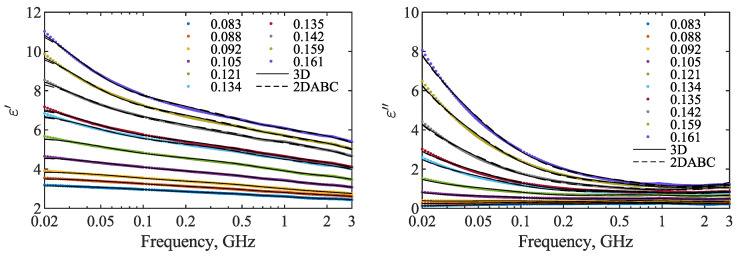
Models 3D (solid black lines) and 2D-ABC (dashed black lines) fitted to the spectra (dots of various colors) of $\varepsilon^{\prime }$ (**left** graph) and $\varepsilon^{\prime \prime }$ (**right** graph) of rapeseed samples of moisture content given in the legend (in g $g^{-1}$). For clarity, for each moisture content, only one spectrum is shown.

The 3D and 2D-ABC dielectric models, described in Section 2.3, were fitted to all obtained spectra. The results obtained for 10 spectra, one for each value of θ, are presented in Figure 4. Both models fitted to all measured spectra well. At frequencies close to 20 MHz, the fitted values were slightly lower than the experimental data, especially for 2D-ABC. In the case of the 3D model, the minimum RMSE of the fit was 0.0072, maximum: 0.0435 and the mean value: 0.0224. 2D-ABC model exhibited slightly higher RMSE, with minimum, maximum and mean values equal to 0.0091, 0.0566 and 0.0283, accordingly.

**Figure 5:**
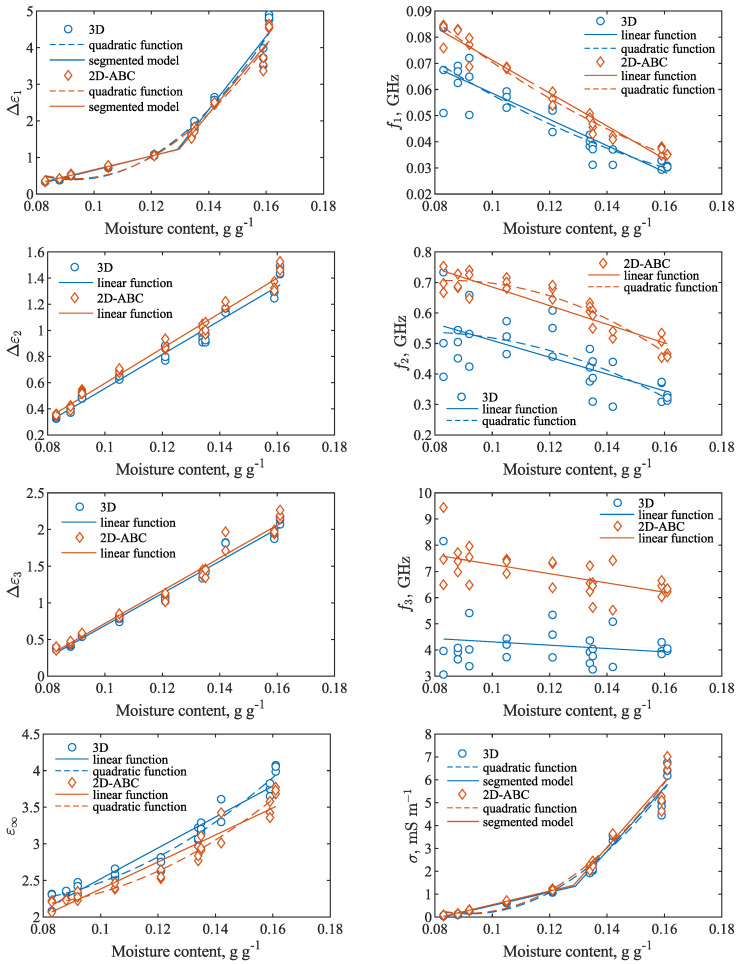
Parameters of the fitted models (Equations (4) and (6)) with respect to the rapeseed moisture content. In the case of the 2D-ABC model, parameters $\varepsilon_{\infty }$, $\Delta \varepsilon_{3}$ and $f_{3}$ were calculated with the use of Equation (7). The parameters’ dependencies on moisture content were modeled by linear, quadratic or segmented functions, as appropriate.

The parameters of the fitted models and their relations with moisture content of the seeds are presented in Figure 5. The 3D and 2D-ABC models virtually produced the same values of the parameters: $\Delta \varepsilon_{1}$, $\Delta \varepsilon_{3}$ and σ. Values of $\varepsilon_{\infty }$ obtained from the 3D model were slightly higher than the values obtained from the 2D-ABC model, while in the case of $\Delta \varepsilon_{2}$, the values obtained from 3D were slightly lower than those from 2D-ABC. All three relaxation amplitudes, as well as $\varepsilon_{\infty }$ and σ, increased with the increase in moisture content. On the other hand, $f_{1}$, $f_{2}$ and $f_{3}$ decreased with the increase in moisture content. Additionally, the values of these relaxation frequencies were higher in the case of the 2D-ABC model than for the 3D model.

The relations between the obtained dielectric parameters and the moisture content of the seeds were modeled by a linear function (Equation (8)), a quadratic function (Equation (9)) or the segmented model (Equation (10)), as appropriate. The results of the fitting, including the determined parameters, RMSE and $R^{2}$ of the fit are presented in Table 3 and Table 4. In the case of $\Delta \varepsilon_{1}$ and σ, the segmented model better fitted the data than the quadratic function. This clearly shows that these parameters increased with the increase in moisture much slower at θ less than approximately 0.13 g $g^{-1}$ than at higher θ values. RMSE and $R^{2}$ were comparable for $\Delta \varepsilon_{1}$ and σ obtained from both 3D and 2D-ABC models. The two different slopes of the relation between these parameters might hint that, in the case of low moisture content, all water is held tightly bound to organic matter particles. Then, after a certain threshold moisture value is reached, additional water molecules are more free to rotate in an external electric field, contributing much more to the overall dielectric permittivity value of the sample than in the case of bound water. A basis for this hypothesis is the difference in dielectric properties between the bound or hydrated water and free water states in the soil and biological systems [1]. Examination of this phenomena in seeds requires further research.

The dependence of $\Delta \varepsilon_{2}$ and $\Delta \varepsilon_{3}$ on the moisture content was successfully modeled by a linear function, while in the case of $\varepsilon_{\infty }$, a quadratic function performed slightly better than the linear one. No large differences between the 3D and 2D-ABC models were obtained for these three parameters. The influence of moisture on $f_{1}$ and $f_{2}$ could be modeled with a linear or quadratic function, but the quadratic function did not perform markedly better than the linear one, with the exception of $f_{2}$ obtained from the 2D-ABC model. For these relaxation frequencies, the relations obtained using the values from the 2D-ABC model exhibited a much better fit, with RMSEs equal to about half the values than in the case of using the 3D model. In the case of $f_{3}$, it occurred that it only slightly depended on the moisture content and significant scattering of the obtained values was observed. A coefficient of determination of less than 0.4 was obtained for a linear $f_{3}\left(\theta \right)$ function using the values from the 2D-ABC model, while in the case of the 3D model, the $R^{2}$ was less than 0.04. The $f_{3}$ values obtained from the 2D-ABC model were more stable, as expected, since this model was developed for fitting to the spectra exhibiting a high-frequency out-of-band pole. Additional measurements in a broader frequency range are required for a detailed analysis of the physical interpretation of the detected dielectric relaxations. Investigation of other dielectric models, including distributed relaxation frequencies, would then also be possible.

## 4. Conclusions

The dielectric properties of whole rapeseeds in the radio and microwave frequency range, knowledge of which is important for the optimization of various production processes and moisture content determination, were determined with good repeatability with the use of a six-channel coaxial transmission-line system connected to a single-port vector network analyzer. The obtained spectra depended on the frequency and moisture content. The $\sqrt{\varepsilon^{\prime }}\left(\theta \right)$ relations were determined to be linear and the frequency dependence of the slope and the intercept was modeled with a fourth-order polynomial equation with respect to the natural logarithm of frequency. Two dielectric models with three poles each were fitted to the spectra in order to analyze their dielectric dispersion and the dependence of the calculated parameters on the moisture content was investigated. The values of the relaxation frequencies exhibited visible scatter, especially in the case of $f_{3}$ and the values obtained from the 3D model. It occurred that the determined $f_{1}$ values are close to the 20 MHz lower limit of the frequency range, especially for samples of the highest θ, while the values of $f_{3}$ exceeded the 3 GHz upper frequency limit. Therefore, the determination of these values should be verified in the extended frequency range in future research. The linear function modeled the relations between the second and third relaxation amplitude and moisture content very well, while for the first relaxation amplitude, the segmented model performed best. This model was also used for σ(θ) relation.

The present research was conducted at room temperature, on a single rapeseed cultivar, and the seeds of diameters from a specific range were selected. Since the experimental approach was tested, further research will involve the measurements of more variable material and at different temperatures in order to obtain relations of practical importance. The presented method does not require special preparation of the material, e.g., grinding the seeds, so it can be used for fast and nondestructive measurements for research and practical purposes.

## Figures and Tables

**Table 1:** Slope *a*, intercept *b*, coefficient of determination $R^{2}$ and root-mean-squared error RMSE of fitting Equation (2), which modeled $\sqrt{\varepsilon^{\prime }}\left(\theta \right)$ relations at given frequencies *f*.

*f*, GHz	*a*	*b*	*R* ^2^	*RMSE*
0.02	18.2	0.268	0.9788	0.076
0.05	14.5	0.560	0.9852	0.051
0.10	12.9	0.678	0.9876	0.041
0.20	11.8	0.734	0.9885	0.036
0.50	11.0	0.760	0.9886	0.034
1.00	10.4	0.773	0.9864	0.035
2.00	9.79	0.776	0.9846	0.035
3.00	9.22	0.801	0.9827	0.035

**Table 2:** Coefficients: $c_{0}$, $c_{1}$, $c_{2}$, $c_{3}$ and $c_{4}$, with their standard errors SE, *t*-statistics and *p*-values, of Equation (3) fitted to the frequency dependence relations of the *a* and *b* parameters of Equation (2) for frequency expressed in GHz.

Parameter	Coefficient	Estimate	SE	*t*-Statistic	*p*-Value
*a*	$c_{0}$	10.3864	0.0022	4715.20	0
	$c_{1}$	−0.8566	0.0032	−263.795	0
	$c_{2}$	−0.0969	0.0029	−33.5964	2 $\times 10^{-125}$
	$c_{3}$	−0.0616	0.0025	−24.9570	2.6 $\times 10^{-87}$
	$c_{4}$	0.0098	0.0005	20.4419	2.1 $\times 10^{-66}$
*b*	$c_{0}$	0.7671	0.0002	3487.80	0
	$c_{1}$	0.0121	0.0003	37.4090	5 $\times 10^{-141}$
	$c_{2}$	0.0094	0.0003	32.4952	9 $\times 10^{-121}$
	$c_{3}$	0.0079	0.0002	31.8873	4 $\times 10^{-118}$
	$c_{4}$	−5.4 $\times 10^{-4}$	5 $\times 10^{-5}$	−11.2541	4.3 $\times 10^{-26}$

**Table 3:** Relations between 3D and 2D-ABC dielectric models’ parameters and moisture content, modeled by Equations (8) and (9), where appropriate. For each fitted equation, the coefficient of determination $R^{2}$ and root-mean-squared error RMSE are given.

Parameter	Model	Linear Function (Equation (8))			Quadratic Function (Equation (9))					
		a	b	$R^{2}$	RMSE	a	b	c	$R^{2}$	RMSE
$\Delta \varepsilon_{1}$	3D	—	—	—	—	864.5	−160.8	7.87	0.9730	0.26
	2D-ABC	—	—	—	—	809.8	−150.8	7.43	0.9726	0.24
$\Delta \varepsilon_{2}$	3D	12.99	−0.74	0.9742	0.06	—	—	—	—	—
	2D-ABC	13.40	-0.74	0.9829	0.05	—	—	—	—	—
$\Delta \varepsilon_{3}$	3D	21.94	−1.50	0.9802	0.09	—	—	—	—	—
	2D-ABC	22.30	−1.51	0.9764	0.10	—	—	—	—	—
$\varepsilon_{\infty }$	3D	21.13	0.41	0.9428	0.15	162.7	−18.44	2.69	0.9680	0.11
	2D-ABC	18.44	0.54	0.9256	0.15	166.1	−21.95	2.87	0.9595	0.11
$f_{1}$, GHz	3D	−0.5013	0.1087	0.8336	0.0064	2.285	−1.057	0.1407	0.8414	0.0064
	2D-ABC	−0.6296	0.1342	0.9654	0.0034	2.662	−1.277	0.1715	0.9732	0.0031
$f_{2}$, GHz	3D	−2.751	0.785	0.4861	0.081	−29.14	4.334	0.376	0.5107	0.080
	2D-ABC	−3.059	0.991	0.8260	0.040	−42.24	7.211	0.399	0.8972	0.031
$f_{3}$, GHz	3D	−6.39	4.95	0.0358	0.95	—	—	—	—	—
	2D-ABC	−17.72	9.04	0.3925	0.63	—	—	—	—	—
σ, mS $m^{-1}$	3D	—	—	—	—	1178	−216.4	10.07	0.9678	0.40
	2D-ABC	—	—	—	—	1207	−220.5	10.23	0.9689	0.40

**Table 4:** Parameters of the segmented model (Equation (10)) fitted to the $\Delta \varepsilon_{1}\left(\theta \right)$ and σ(θ) relations, with $\Delta \varepsilon_{1}$ and σ obtained with the use of 3D and 2D-ABC dielectric models.

Parameter	Dielectric Model	$a_{s1}$	$a_{s2}$	$b_{s1}$	$\theta_{c}$	$R^{2}$	RMSE
$\Delta \varepsilon_{1}$	3D	19.57	100.28	−1.31	0.129	0.9776	0.24
	2D-ABC	18.81	94.48	−1.22	0.130	0.9780	0.22
σ, mS $m^{-1}$	3D	29.09	139.02	−2.41	0.129	0.9717	0.38
	2D-ABC	29.96	142.65	−2.46	0.128	0.9724	0.39

## Data Availability

The data presented in this study are available upon request from the corresponding author.

## References

[1] Feldman Y., Ermolina I., Hayashi Y. (2003). Time domain dielectric spectroscopy study of biological systems. IEEE Trans. Dielectr. Electr. Insul..

[2] Miura N., Yagihara S., Mashimo S. (2003). Microwave Dielectric Properties of Solid and Liquid Foods Investigated by Time-domain Reflectometry. J. Food Sci..

[3] Sosa-Morales M., Valerio-Junco L., López-Malo A., García H. (2010). Dielectric properties of foods: Reported data in the 21st Century and their potential applications. LWT—Food Sci. Technol..

[4] Jha S.N., Narsaiah K., Basediya A.L., Sharma R., Jaiswal P., Kumar R., Bhardwaj R. (2011). Measurement techniques and application of electrical properties for nondestructive quality evaluation of foods—A review. J. Food Sci. Technol..

[5] Trabelsi S., Nelson S.O. (2016). Microwave sensing of quality attributes of agricultural and food products. IEEE Instrum. Meas. Mag..

[6] Zając T., Klimek-Kopyra A., Oleksy A., Lorenc-Kozik A., Ratajczak K. (2016). Analysis of yield and plant traits of oilseedrape (*Brassica napus* L.) cultivated intemperate region in light of the possibilitiesof sowing in arid areas. Acta Agrobot..

[7] Nosenko T., Kot T., Kichshenko V. (2014). Rape Seeds as a Source of Feed and Food Proteins. Pol. J. Food Nutr. Sci..

[8] Vuorela S., Meyer A.S., Heinonen M. (2004). Impact of Isolation Method on the Antioxidant Activity of Rapeseed Meal Phenolics. J. Agric. Food Chem..

[9] Xu B., Wei B., Ren X., Liu Y., Jiang H., Zhou C., Ma H., Chalamaiah M., Liang Q., Wang Z. (2018). Dielectric Pretreatment of Rapeseed 1: Influence on the Drying Characteristics of the Seeds and Physico-chemical Properties of Cold-Pressed Oil. Food Bioprocess Technol..

[10] Niu Y., Rogiewicz A., Wan C., Guo M., Huang F., Slominski B.A. (2015). Effect of Microwave Treatment on the Efficacy of Expeller Pressing of Brassica napus Rapeseed and Brassica juncea Mustard Seeds. J. Agric. Food Chem..

[11] Bansal A.K., Singh P.J., Sharma K.S. (2001). Microwave dielectric measurements in different varieties of rapeseed-mustard seeds in powder form. In. J. Pure Appl. Phys..

[12] Gawrysiak-Witulska M., Rudzińska M., Wawrzyniak J., Siger A. (2012). The Effect of Temperature and Moisture Content of Stored Rapeseed on the Phytosterol Degradation Rate. J. Am. Oil Chem. Soc..

[13] Nelson S.O., Trabelsi S. Measurement of grain and seed moisture and density through permittivity relationships. Proceedings of the 2010 IEEE Instrumentation & Measurement Technology Conference Proceedings.

[14] Boguta A., Majcher J. (2021). The Method of Determining Seed Moisture Based on the Signal Generated by the Piezoelectric Plate. Przegląd Elektrotechniczny.

[15] Jones S.B., Sheng W., Or D. (2022). Dielectric Measurement of Agricultural Grain Moisture—Theory and Applications. Sensors.

[16] Bansal N., Dhaliwal A.S., Mann K.S. (2016). Dielectric characterization of rapeseed (*Brassica napus* L.) from 10 to 3000 MHz. Biosyst. Eng..

[17] Bansal A.K., Singh P.J., Sharma K.S., Kumar S., Kumar P.R. (2001). Dielectric properties of different varieties of rapeseed-mustard oil at different temperatures. Indian J. Pure Appl. Phys..

[18] Ulrych J., Mentlik V. Dielectric properties of sunflower, rapeseed and commonly used mineral oil. Proceedings of the 2016 17th International Scientific Conference on Electric Power Engineering (EPE).

[19] Yu D.U., Shrestha B.L., Baik O.D. (2015). Radio Frequency Dielectric Properties of Bulk Canola Seeds under Different Temperatures, Moisture Contents, and Frequencies for Feasibility of Radio Frequency Disinfestation. Int. J. Food Prop..

[20] Lewandowski A., Szypłowska A., Wilczek A., Kafarski M., Szerement J., Skierucha W. (2019). One-Port Vector Network Analyzer Characterization of Soil Dielectric Spectrum. IEEE Trans. Geosci. Remote Sens..

[21] Vaz C.M., Jones S., Meding M., Tuller M. (2013). Evaluation of Standard Calibration Functions for Eight Electromagnetic Soil Moisture Sensors. Vadose Zone J..

[22] Szypłowska A., Lewandowski A., Jones S.B., Sabouroux P., Szerement J., Kafarski M., Wilczek A., Skierucha W. (2019). Impact of soil salinity, texture and measurement frequency on the relations between soil moisture and 20 MHz–3 GHz dielectric permittivity spectrum for soils of medium texture. J. Hydrol..

[23] MATLAB (2019). 9.6.0.1114505 (R2019a) Update 2.

[24] Szypłowska A., Lewandowski A., Yagihara S., Saito H., Furuhata K., Szerement J., Kafarski M., Wilczek A., Majcher J., Woszczyk A. (2021). Dielectric models for moisture determination of soils with variable organic matter content. Geoderma.

[25] MATLAB Fitlm Function.

[26] Boguta A., Majcher J. Using the physical parameters of rape seeds to assess germination force. Proceedings of the 2017 International Conference on Electromagnetic Devices and Processes in Environment Protection with Seminar Applications of Superconductors (ELMECO & AoS).

